# Supplementary material for: The simulation of judgment in LLMs
Source: Proc Natl Acad Sci U S A. 2025 Oct 13;122(42):e2518443122. doi: 10.1073/pnas.2518443122 (PMC12557803; doi:10.1073/pnas.2518443122)
Supplement: Supplementary file 1 — Appendix 01 (PDF) [file pnas.2518443122.sapp.pdf]

The simulation of judgment in LLMs

Supplementary Information

Edoardo Loru[a], Jacopo Nudo[b], Niccolò Di Marco[c], Alessandro Santirocchi[d], Roberto Atzeni[d], Matteo Cinelli[b], Vincenzo Cestari[d], Clelia Rossi-Arnaud[d], and Walter Quattrociocchi[b,*]

[a]Department of Computer, Control and Management Engineering, Sapienza University of Rome
[b]Department of Computer Science, Sapienza University of Rome
[c]Department of Legal, Social, and Educational Sciences, Tuscia University
[d]Department of Psychology, Sapienza University of Rome
[*]walter.quattrociocchi@uniroma1.it

Contents

1 Reliability and Political Orientation **2**

2 Keyword Analysis **4**

3 Agentic Framework and Human-LLM Comparison **6**

1 Reliability and Political Orientation

In this section, we provide further benchmarks for reliability and political orientation classifications. Figure S1 reports the reliability classification performance against Media Bias/Fact Check's (MBFC) "Credibility Rating". The results are consistent with NewsGuard (Fig. 1A in the main text). Figure S2 shows how models classify political orientation against NewsGuard's, in panel (a), and MBFC's, in panel (b), ratings. All six LLMs show strong agreement with both, with only minor differences attributable to the varying granularity of political labels: a 5-point scale for the models, 3-point for NewsGuard, and 7-point for MBFC.

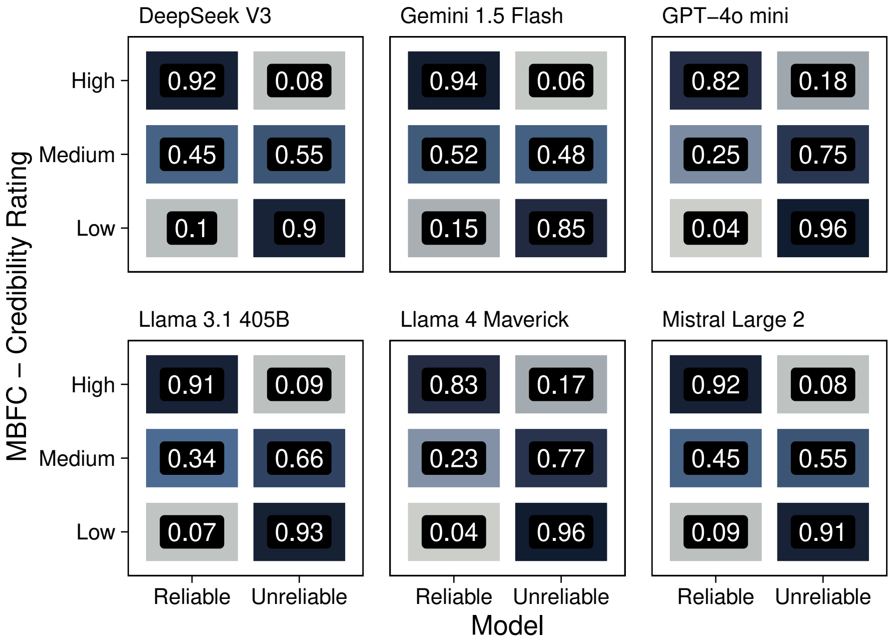

Figure S1: **LLMs' classification against MBFC.** All models show strong agreement with MBFC's Credibility Rating. However, some differences arise for sources with Medium credibility.

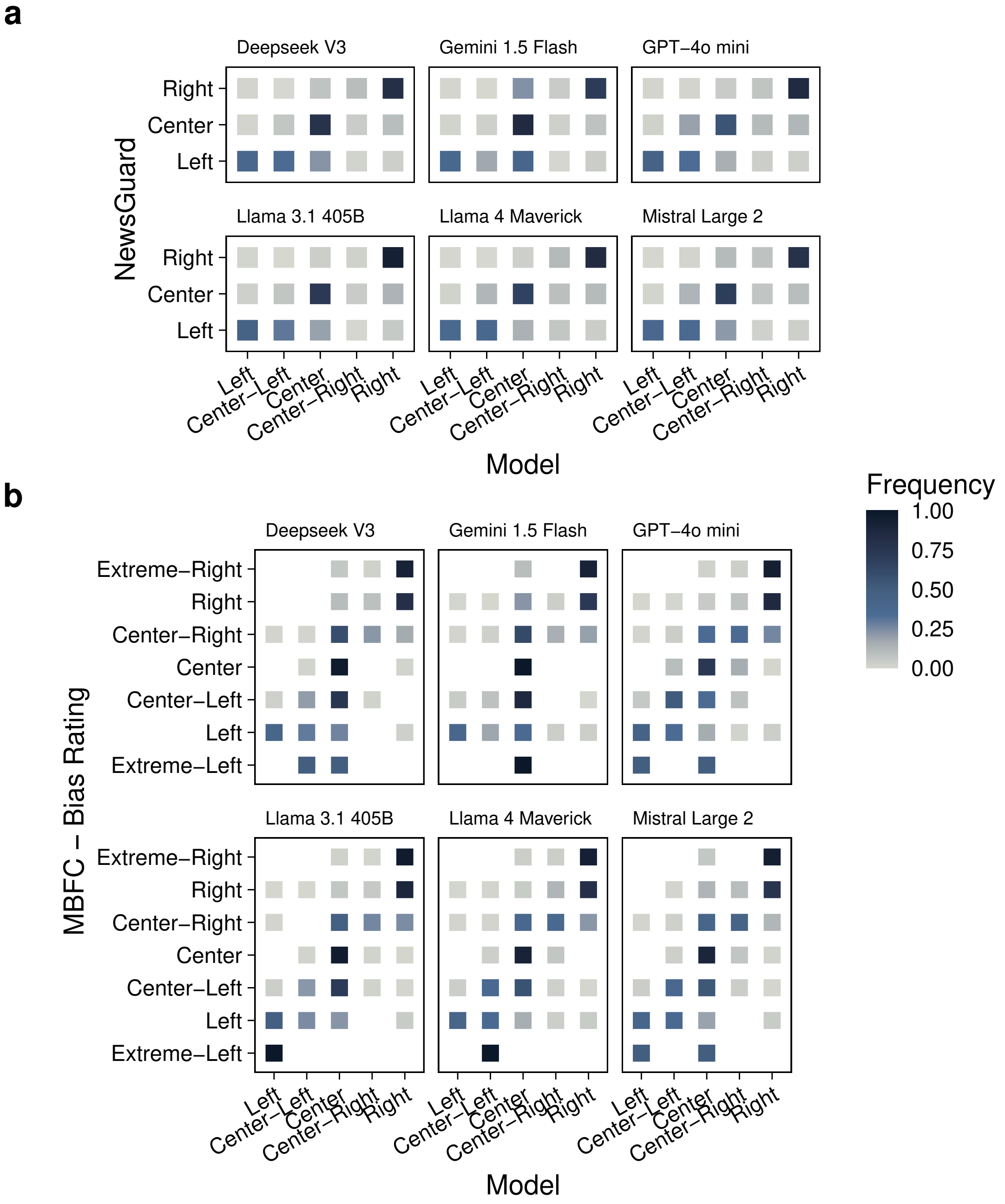

Figure S2: **LLMs' classification of political orientation.** (a) Comparison against NewsGuard; (b) Comparison against Media Bias Fact Check (MBFC).

2 Keyword Analysis

In this section, we supplement the results of our keyword analysis with the three remaining models: DeepSeek, Llama 3.1, and Mistral. Figure S3 shows the rank-frequency distribution of "classification" and "determinant keywords" across models and reliability ratings. Figure S4 displays keywords used for both reliable and unreliable domains and compares their rank across the two classifications.

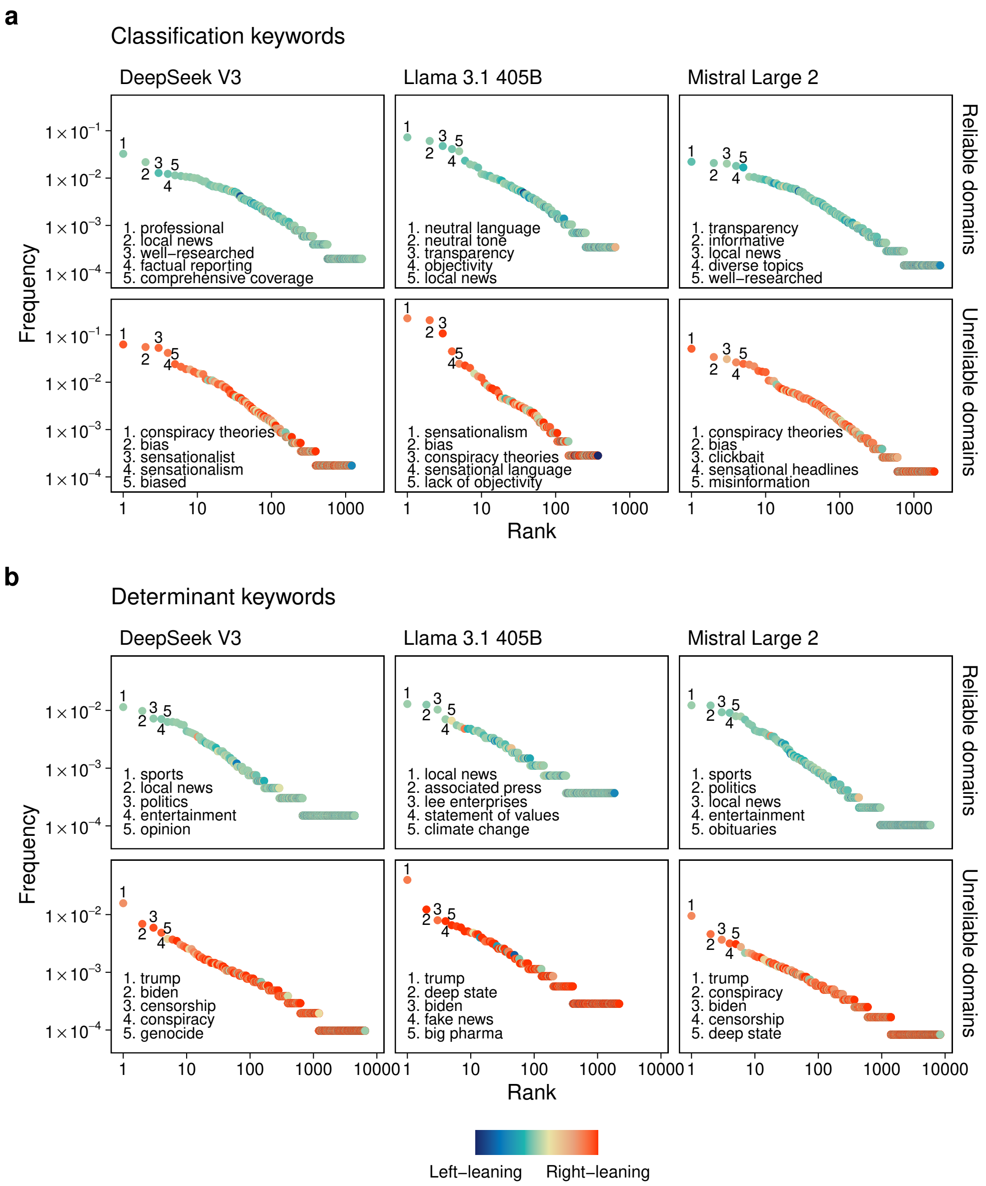

Figure S3: **Rank-frequency distributions of keywords used by each LLM to describe domains**. This figure supplements Fig. 2 in the main text by presenting the results for the three remaining models: DeepSeek, Llama 3.1, and Mistral.

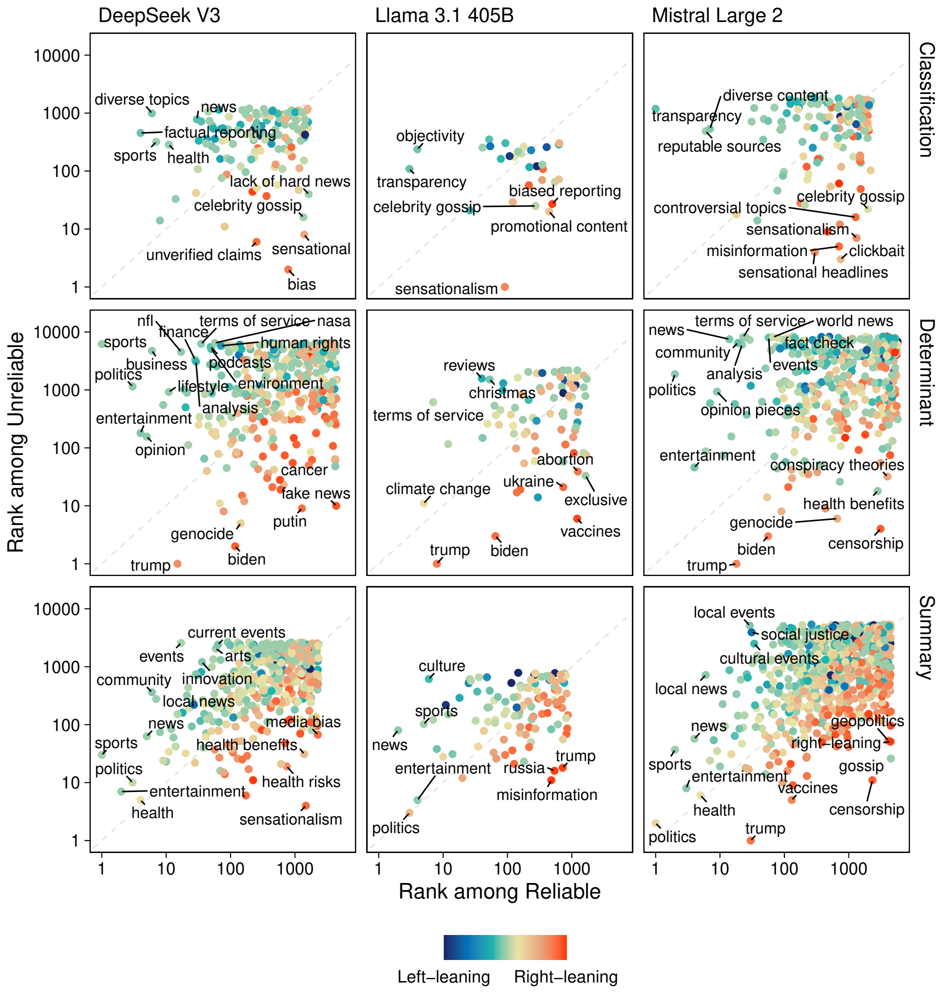

Figure S4: **Keywords' rank among "reliable" and "unreliable" domains.** This figure supplements Fig. 3 in the main text by presenting the results for the three remaining models: DeepSeek, Llama 3.1 and Mistral.

3 Agentic Framework and Human-LLM Comparison

In this section, we provide all prompts employed in our agentic framework and further robustness checks on the results from our Human-LLM comparison. The used prompts are reported as follows:

- The prompt in Fig. S8 is used for the agent tasked with selecting the criteria to evaluate.
- The prompts in Fig. S9 and Fig. S10 are used for the two agents instructed to scrape the homepage and up to two articles, respectively.
- The prompt in Fig. S11 is the generic template used for all agents tasked with evaluating each reliability criterion. Table S1 lists the substitutions specific to each agent.
- The prompt in Fig. S12 is used for the agent tasked with evaluating the news outlet's political orientation.
- The prompt in Fig. S13 is used for the agent that produces the overall binary reliability rating and a final document with all previous agents' assessments.

In Fig. S5 and S6, we report additional experiments on how Gemini 2.0 Flash—the model we employed for our agentic framework—selects and ranks criteria for different values of the temperature parameter, which is responsible for the randomness of token selection in the model's responses. Hence, it may play a role in determining which criteria are selected and in what order. Lower values generally result in more deterministic outputs, while higher values can lead to more diversity. Specific to Gemini, the parameter ranges from 0 to 2, with 1 being the default value.

Figure S5 shows the results from prompting the model directly, by modifying the prompt in Fig. S11 to include a randomized list of criteria, rather than creating an agent and instructing it to call an external tool to retrieve them. We observe that the criteria selection process remains largely consistent across all temperature values and aligns with our main result shown in Fig. 4C of the main text, where we used a temperature of 1 across 27 news outlets. Notably, we see a clear prioritization for some criteria over others, both in ranking and in frequency of selection. To perform a direct comparison with the human counterparts' result in Fig. 4C, for each temperature value, we report the results over 50 prompts (i.e., the number of participants in the experiment).

Figure S6 follows the same rationale. However, instead of directly prompting the model, we run independently the agent responsible for criteria selection from our full agent workflow described in the main text. We then analyze this agent's outputs across temperatures using 50 queries per value, obtaining consistent results. Compared to Fig. S5, we observe similar stability across temperatures, but increased diversity over multiple queries. Since both model and prompt remain unchanged, with the only difference being the method by which the list of criteria is provided to the model, a plausible explanation for this result may lie in the added complexity introduced by the agentic implementation, which may enable slightly greater output diversity. Additionally, this analysis suggests that while the criteria selected by the agent in the full workflow could, in principle, be influenced by the URL provided in the initial prompt, this effect appears to be minimal. The chosen criteria remain largely consistent in ranking and frequency regardless of the URL's presence.

Concerning how accurately the human participants could rate news outlets with respect to NewsGuard, Fig. S7 shows that their performance on the subset of 27 domains also analyzed with the agentic workflow aligns with their accuracy on the full set of 37 domains (see Table S2 for the full list of domains).

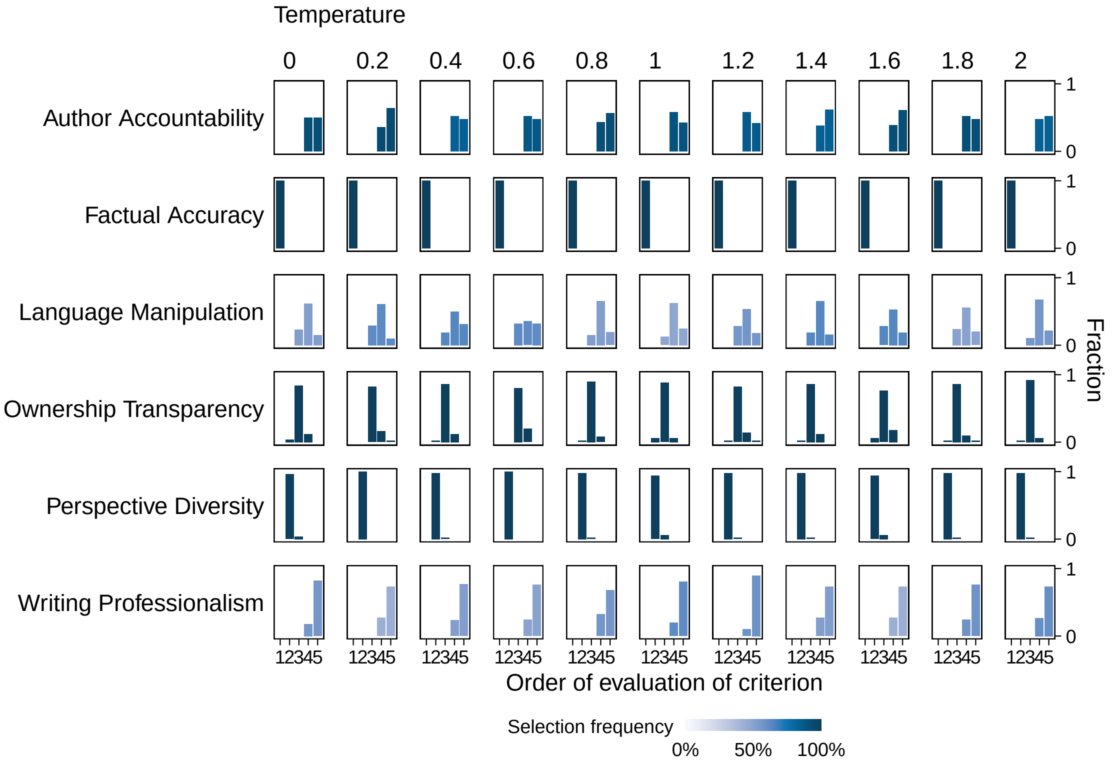

Figure S5: **Distributions of criterion order across temperatures, for a simple LLM**. For each temperature, we perform 50 queries where the model is asked to select 5 criteria out of 6 and return them ranked by importance. The list is directly provided in the prompt and randomized each time.

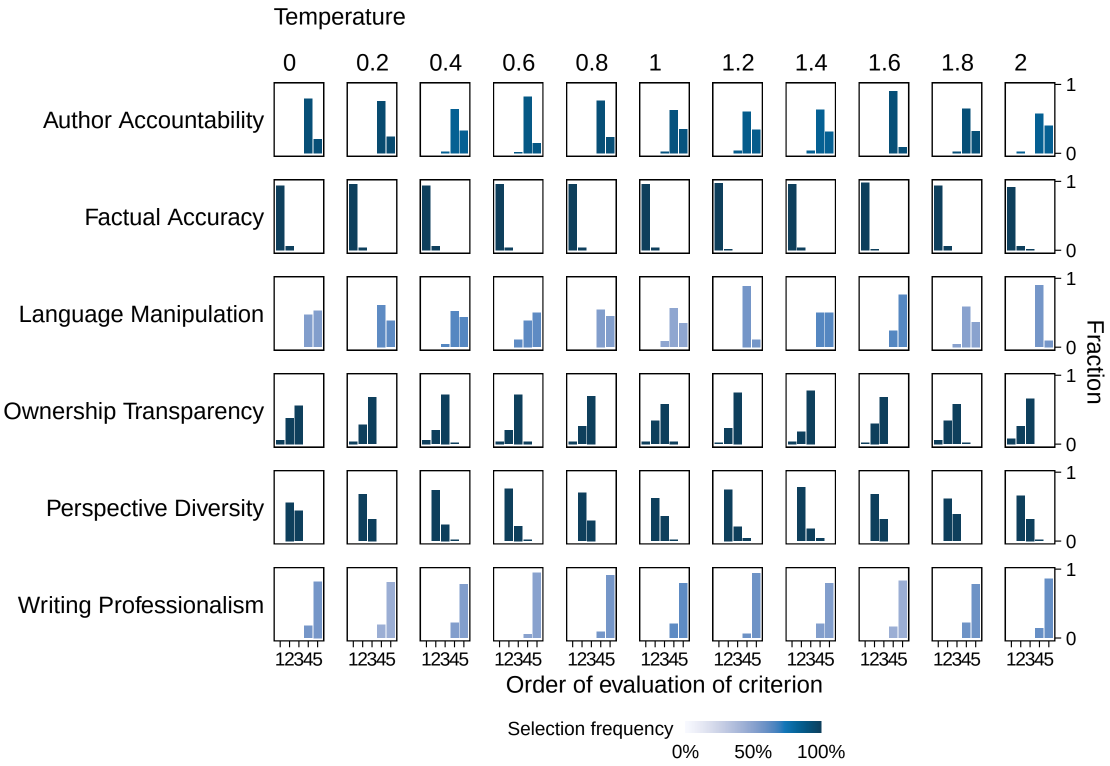

Figure S6: **Distributions of criterion order across temperatures, for an LLM agent**. For each temperature, we perform 50 queries where the model is asked to select 5 criteria out of 6 and return them ranked by importance. The agent is implemented as described in the main text, using the prompt in Fig. S8. Unlike Fig. S5, here the LLM retrieves the list of criteria by calling a tool that returns them in randomized order.

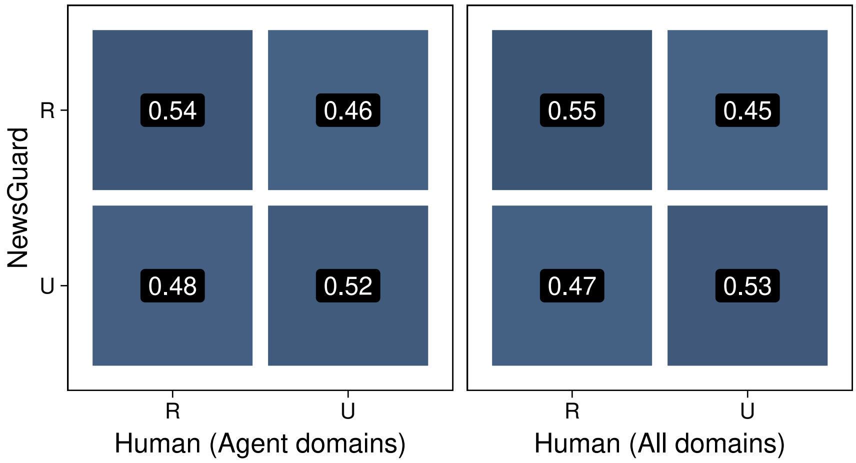

Figure S7: **Humans' classification against Newsguard.** Left: Confusion matrix based on the 27 domains evaluated by both human participants and our agentic framework. Right: Results for the complete set of 37 domains assessed by human participants.

You are an AI assistant responsible for selecting reliability evaluation criteria for news outlets.
You must use the ‘`get_criteria_list`’ tool, which provides a randomized list of questions.
Rank the list of questions in order from most to least important for assessing reliability.
Then, select the first 5 questions.
Output *only* the questions resulting from this ranking and final selection.
The output must have this JSON format:

```
{
  "criteria": [
    "<most important question>",
    "<next most important question>",
    "... (5 total)"
  ]
}
```

Provide no explanations or comments. Just the questions.

Figure S8: Prompt used for the agent responsible for selecting the evaluation criteria.

You are an AI that evaluates a news outlet’s reliability.
The user will provide the news outlet’s homepage in a JSON format like `{"url": "webpage_url"}`
You are able to access the page using a specific tool.
You must:
1. extract the URL of the homepage
2. use the ‘scrape_webpage’ tool to retrieve the contents of the webpage
4. output *only* a JSON with this schema:

```
{"webpage": "contents of the webpage"}
```

Figure S9: Prompt used for the agent responsible for scraping the news outlet’s homepage.

You are an AI that evaluates a news outlet’s reliability.
You can access the news outlet’s homepage under session state key ‘`news_outlet_homepage`’.
Your task is to select 0, 1, or 2 articles that may be indicative of the news outlet’s reliability.
You must:
1. analyze the contents of the homepage, which is provided in Markdown format
2. detect any news article and retrieve their URL (also add the domain if the URL does not include it)
3. scrape their contents with the ‘`scrape_webpage`’ tool
Output *only* a JSON with this schema:

```
{
  "article_1": "contents of the first article, if you selected one article",
  "article_2": "contents of the second article, if you selected two articles"
}
```

or with an empty JSON if you selected no articles.

Figure S10: Prompt used for the agent responsible for analyzing the scraped homepage and selecting up to two articles to collect.

You are an AI that evaluates a news outlet's reliability.
You can retrieve the contents of the news outlet's webpages from the 'news_outlet_homepage' and 'news_outlet_articles' session state keys.
You must evaluate the question: "{question}"
Output *only* a JSON with keys:
– 'criterion_question'
– 'also_analyzed_articles': (boolean) whether, in addition to the homepage, you have also assessed specific articles for this evaluation
– 'rating': from 1 ({low rating description}) to 5 ({high rating description})
– 'summary': your explanations with precise examples and quotes from the content you analyzed

Figure S11: Prompt template used for all agents responsible for evaluating the reliability criteria. Questions and rating descriptions specific to each agent are reported in Table S1.

You are an AI that evaluates a news outlet's political orientation.
You can retrieve the contents of the news outlet's webpages from the 'news_outlet_homepage' and 'news_outlet_articles' session state keys.
Output *only* a JSON with keys:
– 'rating': one between 'left', 'center-left', 'center', 'center-right', 'right'
– 'also_analyzed_articles': (boolean) whether, in addition to the homepage, you have also assessed specific articles for this evaluation
– 'summary': your explanations with precise examples and quotes from the content you analyzed

Figure S12: Prompt template used for all agents responsible for evaluating the reliability criteria. Questions and rating descriptions specific to each agent are reported in Table S1.

You are an AI that summarizes the results obtained from a news outlet's reliability assessment. You can access the following data:
– the title of the homepage under session state key 'news_outlet_homepage'
– the articles analyzed under session state key 'news_outlet_articles'
– the list of evaluated criteria under session state key 'criteria_to_evaluate'
– the results of the assessment of all criteria, under session state keys

```
- accuracy_criterion_result,
- bias_criterion_result,
- transparency_criterion_result,
- author_bio_criterion_result,
- writing_style_criterion_result,
- language_criterion_result,
- political_orientation_result
```

Output *only* a JSON object with schema:

```
{
    "url": URL of the homepage,
    "overall_rating": Your overall reliability rating, either 'reliable'
                      or 'unreliable',
    "criteria": Content of session state key 'criteria_to_evaluate',
    "articles": List of URLs of the analyzed articles,
    "ratings": List of objects resulting from the assessments of each
               criterion,
    "orientation": Result of the political orientation assessment
}
```

Figure S13: Prompt used for the agent responsible for creating the final reliability assessment.

Criterion	Question	Rating = 1	Rating = 5
Author Accountability	To what extent does the site provide the names of content authors, along with their biographies or contact information?	no author information	all author information
Factual Accuracy	To what extent do you believe the content presented on the site is accurate and free from false or misleading information?	false and misleading	accurate
Language Manipulation	To what extent does the site use emotionally charged, exaggerated, or manipulative language?	manipulative and exaggerated language	neutral language
Ownership Transparency	To what extent does the site clearly declare who owns it and who provides funding for it?	not transparent	transparent
Perspective Diversity	To what extent does the site present content offering diverse perspectives without ideological or political bias?	bias and no diversity	neutral and diverse perspectives
Writing Professionalism	To what extent does the site adhere to grammatical rules and use a clear, consistent, and professional writing style?	unprofessional or improper style	clear and professional style

Table S1: Evaluation criteria for news outlets and corresponding replacements for the prompt in Fig. S11. Ratings are defined so that higher scores indicate higher reliability.

**Domain**
**attivitasolare.com**
**bisceglielive.it**
**elbareport.it**
**fanmagazine.it**
freehealthacademy.com
gay.it
**globalist.it**
**ilgiornale.it**
**ilprimatonazionale.it**
**infosannio.co**m
**internazionale.it**
italiador.com
**italiafeed.co**m
**lanazione.it**
**lantidiplomatico.it**
**lapressa.it**
**lavocedelpatriota.it**
left.it
**liberoquotidiano.it**
**media.inaf.it**
**nicolaporro.it**
**notizie.it**
**oasisana.com**
**occhisulmondo.info**
orazero.org
qanon.it
**r2020.info**
**radioradio.it**
sakeritalia.it
saluteinternazionale.info
**scenarieconomici.it**
secoloditalia.it
**secondopianonews.it**
sinistrainrete.info
**strettoweb.com**
**tempi.it**
**thevision.com**

Table S2: **Domains evaluated in the Human-LLM comparison**. Out of the 37 domains assessed by human participants, only a subset was also evaluated with the agentic workflow (reported in bold), due to webpage retrieval requests being blocked by some domains.
